# Pullulan-1-Ethyl-3-Methylimidazolium Tetrafluoroborate Composite as a Water-Soluble Active Component of a Vibration Sensor

**DOI:** 10.3390/s24041176

**Published:** 2024-02-10

**Authors:** Giovanna Di Pasquale, Salvatore Graziani, Antonino Pollicino, Carlo Trigona

**Affiliations:** 1 Dipartimento di Scienze Chimiche (DSC), University of Catania, Viale Andrea Doria 6, 95125 Catania, Italy; giovanna.dipasquale@unict.it; 2 Dipartimento di Ingegneria Elettrica Elettronica e Informatica (DIEEI), University of Catania, Viale Andrea Doria 6, 95125 Catania, Italy; carlo.trigona@unict.it; 3 Dipartimento di Ingegneria Civile e Architettura (DICAr), University of Catania, Viale Andrea Doria 6, 95125 Catania, Italy

**Keywords:** pullulan, electrospinning, imidazolium ionic liquid, piezoionic composite, vibration sensor

## Abstract

In recent years, the issue of electronic waste production has gained significant attention. To mitigate the environmental impact of e-waste, one approach under consideration involves the development of biodegradable electronic devices or devices that dissolve in the environment at the end of their life cycle. This study presents results related to the creation of a sensor that effectively addresses both criteria. The device was constructed using a composite material formed by impregnating a pullulan membrane (a biodegradable water-soluble biopolymer) with 1-Ethyl-3-Methylimidazolium tetrafluoroborate (a water-soluble ionic liquid) and coating the product with a conductive silver-based varnish. Capitalizing on the piezoionic effect, the device has demonstrated functionality as a vibration sensor with a sensitivity of approximately 5.5 × 10^−5^ V/mm and a resolution of about 1 mm. The novelty of this study lies in the unique combination of materials. Unlike the use of piezoelectric materials, this combination allows for the production of a device that does not require an external potential difference generator to function properly as a sensor. Furthermore, the combination of a biopolymer, such as pullulan, and an ionic liquid, both readily soluble in water, in creating an active electronic component represents an innovation in the field of vibration sensors.

## 1. Introduction

The increase in e-waste production is a concerning trend that has been observed because of the proliferation of electronic devices in our daily lives. People own more electronic devices than ever before. Moreover, the pace of technological innovation and product development is accelerating. As a result, people are replacing their electronic devices more frequently to keep up with the latest features and capabilities, leading to a higher turnover of electronics. In addition, some manufacturers design products with planned obsolescence in mind, making it difficult or expensive to repair or upgrade devices. This encourages consumers to discard and replace devices rather than repair them. Many people are also not fully aware of the environmental and health hazards associated with improper e-waste disposal. This lack of awareness can result in electronic devices being disposed of inappropriately [1].

To address the increasing e-waste problem, it is essential to implement strategies such as extended producer responsibility, recycling programs, consumer education, and eco-friendly product design. Crafting electronic components that are environmentally friendly is essential for mitigating the ecological footprint of electronics from their production phase up to disposal. Some key principles and practices for designing electronic components in an environmentally friendly way need a multimodal approach that starts with conducting a lifecycle assessment of electronic components to understand their environmental impact from cradle to grave and identifying areas for improvement. Among others, this includes the optimization of energy efficiency and modular design of the components, the selection of materials with a lower environmental impact that are easy to recycle at the end of a component’s life, and avoiding the use of hazardous materials, such as lead, mercury, and cadmium, in component manufacturing [2].

In this context, a new approach has been proposed in the last ten years, which can be summarized by the term “transient electronics”. Transient electronics refers to a field of electronics that involves the development and use of electronic devices and components that have a limited and controlled lifespan. The development of transient electronics involves using materials and components that are designed to break down in a controlled and environmentally-friendly manner. This field is still in the research and development stage, but it holds promise for addressing various technological challenges while minimizing long-term environmental impacts [3].

Electronic devices made with organic and/or water-soluble biopolymeric materials as the main components, both active and passive, would represent a significant step forward in meeting the requirements associated with the transient electronics approach.

In this scenario, the development of electronic devices, such as vibration and deformation sensors, using polymeric membranes with piezoelectric or piezoionic characteristics would move in the desired direction.

Polymeric membranes demonstrate considerable promise in advancing vibration sensor technology, boasting heightened sensitivity, flexibility, and multifunctional capabilities. The incorporation of cutting-edge technologies and the continual development of innovative sensing systems serve to amplify the prowess of polymeric membranes in various vibration sensor applications. Their utilization in sensor applications has garnered attention across various fields, including energy harvesting and, more generally, electronic engineering. Di Pasquale et al. highlighted the broad applicability of all polymeric transducers for energy harvesting [4]. Chen showcased a vibration sensor of remarkable sensitivity and full flexibility, featuring a suspended sensing membrane designed with channel cracks [5]. This design provides exceptional capabilities for dynamic vibration and acceleration monitoring. In a separate experiment, Li demonstrated a versatile chemical sensing platform using a dual-resonant infrared plasmonic perfect absorber, enabling on-chip detection of poly(ethyl cyanoacrylate). This underscores the potential of polymeric membranes in vibration detection and materials characterization [6]. Meanwhile, Guo conducted impactful experimental research on pretension rectangular membrane structures, underscoring the significance of accurate vibration measurement achievable with polymeric membrane sensors [7].

The use of polysaccharides or biopolymers in developing transient electronic devices is an innovative approach that has gained attention in recent years [8,9,10]. Polysaccharides, which are long chains of sugar molecules, and biopolymers, which are natural polymers derived from living organisms, offer, when incorporated into transient electronics, several advantages such as biodegradability, environmental friendliness, biocompatibility, customizable properties, and wide availability. In fact, polysaccharides and biopolymers are inherently biodegradable and can be designed to break down naturally and harmlessly over time. This property aligns with the concept of transient electronics, where the devices should disintegrate or dissolve after a specific period. Moreover, the use of biopolymers reduces the environmental impact associated with electronic waste. Due to their biocompatibility, they are suitable for use in medical implants and healthcare applications. Polysaccharides and biopolymers can also be modified and engineered to have specific properties, such as mechanical strength, flexibility, or degradation rate [11]. Finally, many polysaccharides and biopolymers are readily available from renewable sources. The potential of polysaccharides and polysaccharide nanocomposites in electronic appliances and their relevance in the field of mechanoelectrical sensors was emphasized in several papers [12,13,14,15].

Among polysaccharides, pullulan (PUL), a linear glucosic polysaccharide derived from the polymorphic fungus Aureobasidium pullulans, has extensive applications across diverse sectors, including food additives, environmental remediation, and pharmaceutical formulations [16]. This distinctive biopolymer, boasting numerous patented applications, has been explored for its potential in areas such as food packaging, biomedical materials, and shape memory polymers due to its elevated molecular weight and abundant hydroxyl content. In the realm of sensor technology, PUL has played a pivotal role in the development of various sensors, including paper sensors for bacterial detection and serum lactate dehydrogenase detection [17,18]. In a study by Prosini et al. [19], PUL was employed as a binder in supercapacitors, utilizing carbon electrodes derived from waste pepper seeds. Expanding its utility, PUL has been incorporated into PUL-ionic liquid-based supercapacitors, as highlighted by Poli et al. [20], emphasizing its role in creating easily disposable devices. PUL has emerged as a notable candidate for diverse applications in electronic devices, capturing attention for its unique attributes. It holds significance across multiple domains within electronic and measurement sciences, particularly standing out in printed electronics due to its distinct characteristics. For instance, PUL has been integrated into nanocomposites with minimal loading of graphene oxide, showcasing exceptional oxygen barrier capabilities crucial for electronic packaging [21]. In another application, PUL has been instrumental in creating high-k polymer cyanoethylated PUL (CEP) thin films for use in organic field-effect transistors. These films exhibit advantages such as low-voltage operation and a high dielectric constant [22]. Additionally, PUL plays a role in transparent circuit boards and foldable conductive nanopaper for LED lighting, underscoring its potential in electronic circuits and devices [23]. Finally, its biodegradation involves the action of enzymes produced by microorganisms, primarily bacteria and fungi, which break down the polymer into simpler compounds [24]. The degradation process typically includes enzymatic hydrolysis of the glycosidic bonds within the pullulan structure. Microorganisms secrete enzymes such as pullulanase, amylase, and glucoamylase, which target the glycosidic linkages between the glucose units in pullulan. These enzymes catalyze the hydrolysis of pullulan into smaller oligosaccharides and eventually into individual glucose molecules. Subsequently, microorganisms utilize these simpler sugars as a source of energy and carbon for their growth. The appeal of PUL extends beyond its electronic performance, with its biodegradability, renewability, water solubility, and non-toxicity positioning it as an attractive candidate for the development of eco-friendly biodegradable electronics.

In recent years, the significance of ionic liquids (ILs) has grown, prompting an increasing number of scientists and engineers to explore potential applications for these liquids due to their distinctive physical and chemical properties. ILs possess a distinctive array of properties that position them as crucial candidates for various energy-related applications. Combinations of cations and anions with low volatility, coupled with high electrochemical and thermal stability, as well as ionic conductivity, open avenues for designing ideal electrolytes in batteries, supercapacitors, actuators, dye-sensitized solar cells, and thermo-electrochemical cells [25], and for their use in gel polymer electrolytes for flexible lithium-ion polymer batteries [26]. Moreover, due to their high solubility in water, they possess characteristics that fall within the realm of transient electronics. Some of our previous works investigated the role of ILs in mechanoelectrical transducers based on bacterial cellulose, further highlighting the potential of polysaccharide-based materials in mechanoelectrical sensing [27,28,29].

The term “piezoionic effect” specifically refers to a phenomenon observed, for example, when there are mobile ions in a polymer matrix. In the case of polymer materials, this behavior can be obtained using an ionomer, such as nafion [30], or through a composite where a polymer matrix contains an ionic liquid. In materials exhibiting this effect, mechanical deformation or stress can induce the movement of ions, leading to ionic conductivity. While the piezoionic effect may not be as universally recognized as traditional piezoelectric materials, recent research [31] has brought attention to its potential applications. The piezoionic effect holds promise in energy harvesting, where materials displaying this effect could convert mechanical energy (such as induced vibrations and movements) into electrical energy. Additionally, these materials can find applications in sensors and transducers, as they can be utilized to create sensors that are responsive to mechanical stimuli. Furthermore, materials with the piezoionic effect can serve as actuators in microfluidic systems, valves, or other devices requiring both mechanical movement and ionic transport. Hybrid devices that combine piezoelectricity and ionic conductivity may open avenues for the development of unique and multifunctional materials in materials science and engineering. It is crucial to note that ongoing research into the piezoionic effect reveals that the specific properties and applications of these materials may vary based on the chosen polymer matrix, ionic liquid, and processing techniques. Researchers are actively exploring the potential of piezoionic materials across various fields, and as they delve deeper, the practical applications of these materials may continue to evolve. It is essential to recognize that the understanding of the piezoionic effect is a dynamic area of study, and ongoing investigations will contribute to refining our knowledge of these materials and expanding their practical utility.

The novelty of our study presented in this work lies in the unique combination of materials. Unlike the use of piezoelectric materials, this combination allows for the production of a device that does not require an external potential difference generator to function properly as a sensor. Furthermore, the combination of a biopolymer (such as pullulan, known for its biocompatibility and biodegradability) and an ionic liquid, both readily soluble in water, in creating an active electronic component represents an innovation in the field of vibration sensors. This innovation holds promising prospects for addressing issues related to e-waste and in the development of new composites that can be categorized within the field of transient electronics.

In this study, we present the results of our investigation on sensors obtained by utilizing a readily available biopolymer employed to produce membranes through electrospinning. These membranes were subsequently infused with an ionic liquid (IL) and coated with a conductive varnish containing silver. The resulting composite materials underwent comprehensive characterization, including morphological, chemical, and electromechanical analyses.

## 2. Materials and Methods

PUL used for the electrospinning process was furnished by Alpha Aesar and was used without purification. 1-Ethyl-3-Methylimidazolium tetrafluoro borate (EMIMBF_4_) was purchased from Alpha Aesar. The conductive silver-based varnish spray used as electrode was furnished by Tifoo. Electrospinning was carried out by using a commercially available apparatus (Starter Kit 40 kV Web, Linari srl, Pisa, Italy) consisting of a high-voltage power supply, a syringe pump, a syringe, a stainless steel blunt-ended needle connected with the power supply electrode, and a grounded drum collector covered by aluminum foil. PUL electrospun scaffolds were produced by dissolving the PUL at a concentration of 23% *w*/*v* in a solution of HPLC-grade water. The polymer solution was poured into a 20 mL syringe equipped with 21-gauge needle (inner diameter = 0.8 mm). A voltage of 18 kV was applied by the high-voltage power supply of the Linari starter kit. The perpendicular distance between the syringe tip and the grounded drum collector (50 rpm) was set at 9 cm. The flow rate of 0.9 mL/h was controlled by the pump speed. The electrospinning process was carried out at 25 °C and 52% of relative humidity.

To fabricate the devices, electrospun PUL membranes (2 cm × 10 cm) were cut, dried in an oven at 40 °C, soaked with EMIMBF_4_ for 24 h, and left again in an oven for 24 h at 40 °C. PUL/EMIMBF_4_/Ag devices were fabricated by twice spraying a conductive varnish on both sides of the membrane. After every deposition, the membranes were dried in an oven for 5 min (T = 40 °C).

### Characterization Methods

The weight percentage content of EMIMBF_4_ and silver-based varnish were determined by using an analytical balance.

Thermogravimetric analyses (TGA) were carried out via a Shimadzu model DTG-60 instrument. TGA curves were recorded at a heating rate of 20 °C min^−1^, in static air atmosphere, from 35 °C to 700 °C. Analyzed sample mass varied between 8.0 mg and 11.0 mg. Temperature and weight calibrations were performed following the procedure reported in the instruction manual of equipment [32] and using the following as standard materials: indium (NIST SRM 2232), tin (NIST SRM 2220), and zinc (NIST SRM 2221a) for temperature; indium (NIST SRM 2232) for heat flow; and a set of exactly-weighed samples supplied by Shimadzu for the weight. All calibrations of equipment were repeated every 2 weeks.

Scanning Electron Microscopy (SEM) micrographs were obtained using a SEM EVO (Zeiss, Cambridge, UK) instrument equipped with an energy dispersive X-ray microanalysis (EDX) facility. The analyses were performed by setting a high electron beam voltage (EHT) of 20 kV and using a LaB6 (Lanthanum Hexaboride) emitter as the electron source. To carry out the SEM analysis, the samples were gold-sputtered with a thin gold film deposited through a sputtering process carried out using an Agar Sputter Coater AGB7340 spray coating machine (Assing, Italy).

A Perkin Elmer Spectrum 100 spectrometer was employed in the determination of the Fourier Transform Infrared (FTIR) spectra. Analyses were carried out on samples at r.t., without any preliminary treatment, using a universal ATR sampling accessory, from 4000 to 650 cm^−1^, with a resolution of 2.0 cm^−1^. The results are an average of three experimental runs to test the results’ reproducibility. Analysis was performed at room temperature, directly on the sample, without any preliminary treatments. Calibration was carried out following the procedures described by Perkin Elmer Spectrum 100 Series User’s Guide. Dynamical mechanical analysis (DMA) was carried out using a 2000 TA DMA produced by Triton Technology Ltd. (London, UK). The frequency dependence of the membrane modulus was evaluated, in single cantilever mode, by applying a sinusoidal force to a rectangular sample, in the range 0.1–50 Hz, at the working temperature of about 25 °C. The calibration was carried out before every measurement session via the automatic procedure present in the instrument control software.

XPS spectra were recorded with a VG Microtech Ltd. (Uckfield, UK) with a CLAMII analyser. The X-ray source (Mg Kα, 1253.6 eV) worked at 200 kV and 10 mA at a pressure < 2 × 10^−8^ Torr, pass energy of 100 eV, and take-off angle of 45°. Binding energies were referenced to the C—H level at 285.0 eV of the so-called adventitious carbon (AdC).

The investigation into the piezoionic PUL sensor’s response to mechanical vibrations was conducted on rectangular samples (1.5 cm × 5 cm – 500 μm thick) with a suitable experimental setup. This setup comprised instruments to gauge the sensor’s electrical signals under vibration and a shaker to apply controlled kinetic movement. To analyze the mechanoelectrical transduction behavior of the PUL-based device, the utilized configuration includes the following:

- A Vibration Test System TV 51110 with a BAA 120 power amplifier (max voltage RMS 22 V and max current RMS 5.5 A, with a nonlinear harmonic distortion factor < 0.05%). This system induced motion in the pullulan-based sensor configured as a cantilever beam. Its frequency range spanned 2–7000 Hz, with the main resonance frequency exceeding 6500 Hz and a maximum displacement peak–peak of 13 mm.
- A Keysight Technologies 33220A function/arbitrary waveform generator (Keysight Technologies Italy S.r.l., Milano, Italy) used to drive the power amplifier BAA 120, delivering known vibration levels. It utilized direct digital synthesis techniques to produce stable, accurate output signals with low distortion, including waves with fast rise and fall times up to 20 MHz and linear ramp waves up to 200 kHz.
- Two Baumer 12U6460/S35A laser sensors (Baumer Italia S.r.l. Assago MI, Italy) with an approximate resolution of 2 µm and programmable sensitivity. These sensors measured displacements at both the anchor and sensor tip, enabling estimation of sensor deformation by analyzing the difference between the two displacements.
- An accelerometer, model PCB333B40-SN51174 (PCB Piezotronics, Depew, NY, USA), placed on the shaker’s moving plate, functioning as the feedback element. This accelerometer presents a resolution of about 0.0005 m/s² rms and a sensitivity of 51 mV/m/s².
- An Agilent Infiniium MSO9064A, a 600 MHz 10 GSa/s 4 Plus 16 Channel Mixed Signal Oscilloscope (Agilent, Santa Clara, CA, USA), equipped with 8-bit resolution and an input sensitivity up to 5 V/div. It was utilized to acquire the output voltage from the pullulan-based sensor, the two lasers, and the feedback accelerometer.

Calibration of all the devices was carried out following the procedures described by the manufacturers.

## 3. Results and Discussion

**Figure 1:**
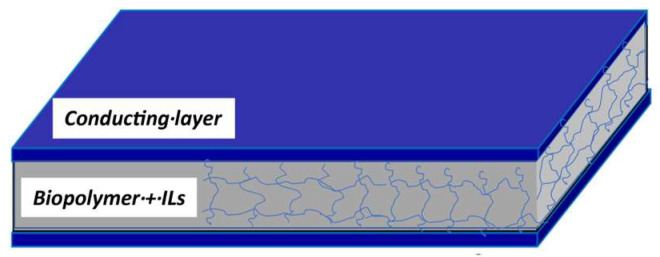
Three-layer structure of the studied device.

Our research aimed to develop a vibration detection device utilizing the piezoionic effect generated through a composite PUL/ionic liquid. The realized device has a three-layer structure formed by a PUL electrospun scaffold immersed in EMIMBF_4_ and positioned between two silver-based electrodes (Figure 1).

Through weighing the samples at various preparation stages, the weight percentages were determined as follows: 44% PCL, 52% EMIMBF_4_, and 4% conductive varnish.

**Figure 2:**
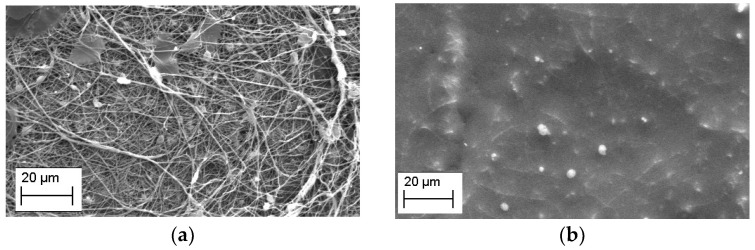
SEM micrographs showing the morphology of the membrane when (**a**) electrospun and (**b**) soaked with the EMIMBF_4_ IL.

To create the PUL scaffolds, the polysaccharide was dissolved at a 23% *w*/*v* concentration in an HPLC-grade water solution. Electrospinning outcomes were influenced by several factors, categorized as solution properties (polymer concentration, molecular weight, viscosity, surface tension, solution vapor pressure, electrical conductivity, and dielectric constant), process parameters (flow rate, spinning voltage, electrode configuration, and electrode distance), and environmental conditions (temperature and humidity). Our experiment utilized water as a solvent, a syringe tip-to-grounded drum collector distance of 9 cm, and a 0.9 mL/h flow rate, resulting in a scaffold featuring nanofibers having an average diameter of 300 nm, rare beads, and some regions where the slow evaporation of the solvent produced flat polymer precipitate, as depicted in the SEM micrograph (Figure 2a). Subsequent membrane soaking with EMIMBF_4_ induced a morphological transformation evident in the SEM micrograph (Figure 2b). Although a few fibers remained visible, the IL presence manifested as a continuous phase, wetting the fibers and permeating the membrane’s pores.

Since the supplier did not provide information on the exact composition of the conductive paint, we analyzed the composition of the electrodes, once deposited on both sides of the sample, through X-ray Photoelectron Spectroscopy (XPS).

**Figure 3:**
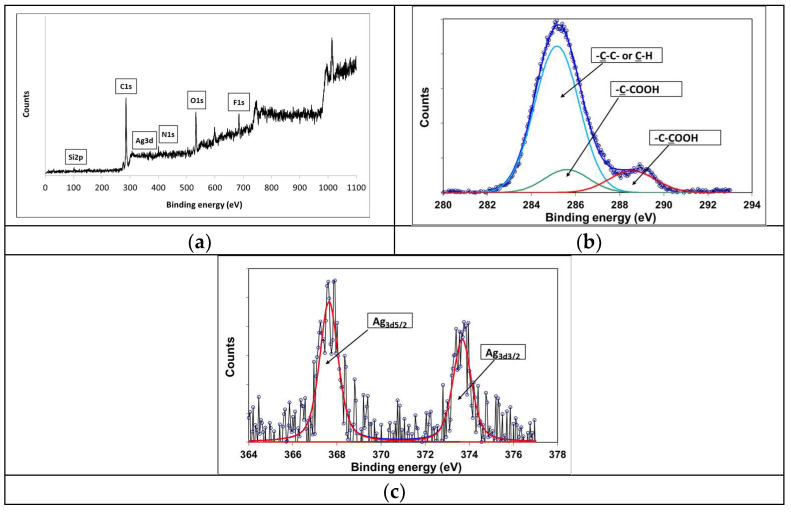
(**a**)Wide-scan XPS spectra of the surface of the device, (**b**) narrow-scan of C_1s_ region, and (**c**) Ag_3d_ region.

The wide-scan in Figure 3a evidenced that carbon, oxygen, nitrogen, silver, fluorine are present on the electrode surface, along with silicon as a contaminant. Analyzing the C_1s_ peak (centered at about 285 eV) shown in Figure 3b, the presence of the peak component centered at about 289.1 eV testified that the polymer matrix of the conductive varnish is an acrylic polymer [33]. The peaks centered at about 400 (N_1s_) and 685 eV (F_1s_) originate from the aforementioned elements emerging on the surface of the IL. The peak centered at about 368 and 374 eV (Figure 3c), due to Ag_3d_ core level, confirm that the conductivity of the varnish is due to presence of silver particles dispersed in a matrix [34].

**Figure 4:**
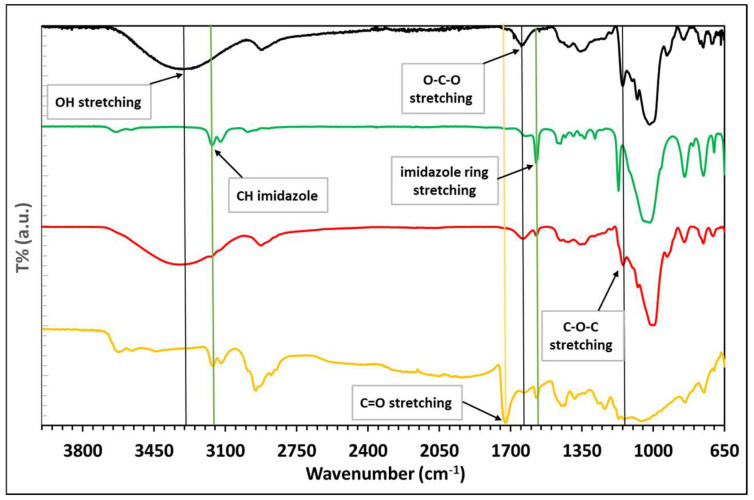
ATR FTIR spectra of PUL (black), EMIMBF_4_ (green), PUL/EMIMBF_4_ (red), and PUL/EMIMBF_4_ /Ag (yellow) samples.

The ATR FTIR, registered in the range 4000–650 cm^−1^ (Figure 4), showed that the core device’s present IR characteristic peaks of PUL (strong absorption at around 3300 cm^−1^) were caused by the –OH stretching vibration of polysaccharides and the characteristic signals obtained at 2918, 1647, and 1148 cm^−1^ were attributed to C–H, O–C–O, and C–O–C stretching [35], while EMIMBF_4_ bands (at 3165 and 3124 cm^−1^), assigned to the C-H of the imidazole ring (the bands at 1576 and 1457 cm^−1^), are due to the imidazole ring skeleton stretching vibration and the broad peak at 1017 cm^−1^ was due to the C-H of the imidazole ring stretching vibration. The ATR spectrum of the electrode confirmed the acrylic nature of the polymer matrix of the varnish as it can be deduced from the presence of a strong absorption band at 1725 cm^−1^, which can be assigned to C=O stretching vibrations of the carboxylic functional groups.

**Figure 5:**
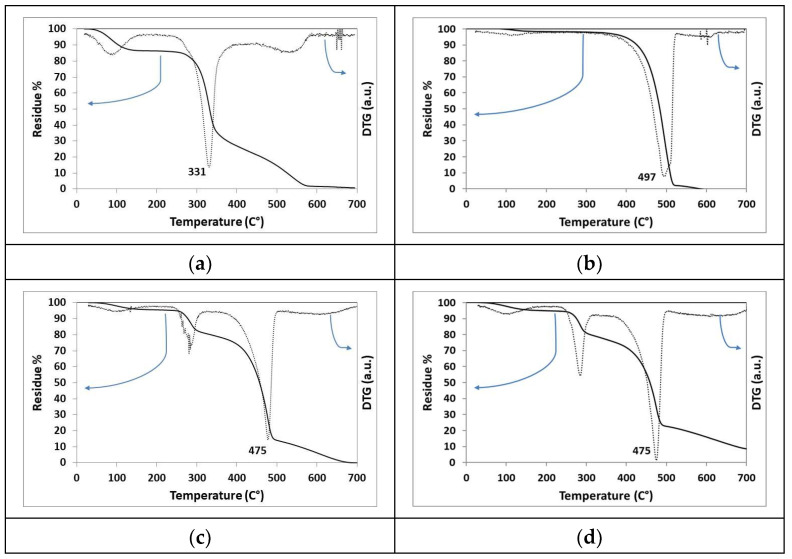
TGA (continuous line) and DTG (dashed line) plots of the PUL membrane as (**a**) electrospun, (**b**) EMIMBF_4_, (**c**) PUL membrane soaked with EMIMBF_4_, and (**d**) soaked membrane coated with silver-based electrodes.

Concerning the thermal stability of the device, some useful information can be drawn comparing TGA curves of electrospun PUL membrane, EMIMBF_4_, PUL/EMIMBF_4_ composite, and PUL/EMIMBF_4_/Ag device. In both PUL/EMIMBF_4_ composite and PUL/EMIMBF_4_/Ag device thermograms, the DTGA curves clearly show that the interaction between PUL and EMIMBF_4_ promotes their thermal degradation. In fact, in PUL thermogram (Figure 5a), where the temperature dependence of the PUL weight loss is plotted, the DTG curve displays, following the peak related to the water loss centered at 90 °C, one main degradation process with the maximum of the weight loss rate at 331 °C and a shoulder at a higher temperature of ca. 535 °C. In the presence of EMIMBF_4_, the main degradation peak shifts to a lower temperature (285 °C) (Figure 5c,d). In the same way, while the DTG curve of pure EMIMBF_4_ shows a peak centered at 497 °C (Figure 5b), the presence of PUL determines a shifting of this peak to 475 °C (Figure 5c,d).

Resonant beams in pinned configuration are widely proposed in the literature for realizing vibrating sensors. In such structures, the measurand changes the resonant frequency, whose value is used to estimate the corresponding measurand value [36]. The investigated frequency range has been fixed in such a way as to contain the mechanical resonant frequency of the investigated device. The mechanical sensor obtained is anticipated to operate within the low-frequency range. Consequently, our aim was to investigate the viscoelastic properties of the devices within the 0.1–50 Hz range at the operating temperature of 25 °C. Measurements of the storage modulus (E′) and the loss modulus (E″) were acquired and compared to trace the evolution of mechanical properties from the raw PUL membrane to the final device. The results obtained at frequencies of 1, 10, and 50 Hz are presented in Table 1.

From the data reported in Table 1, it can be observed that the values of E′ and E″ are strongly influenced by the presence of EMIMBF_4_. In the case of E’, a drop of one order of magnitude is observed, and this result can be rationalized in terms of the PUL–IL interaction that determines a kind of plasticization effect. Furthermore, with the increase in frequency, as expected, the values of E′ and E″ tend to slightly increase, indicating the absence of peaks related to the activation of dissipative phenomena.

**Scheme 1:**
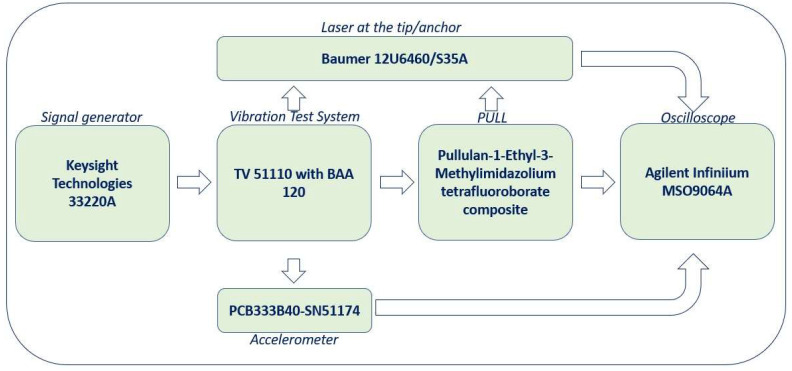

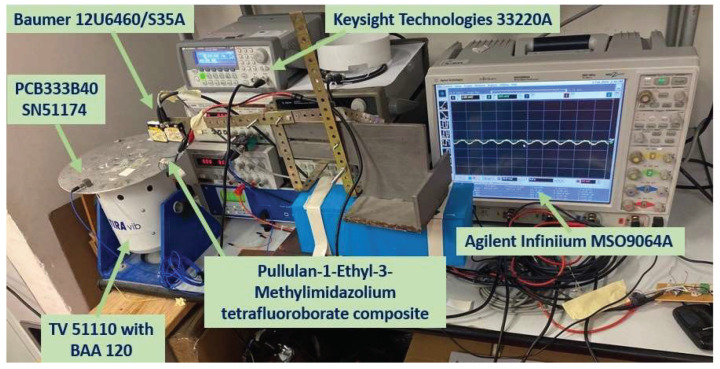
Schematic description of the entire setup and measurement methods for determining the response of the piezoionic pullulan sensor to deformation.

Concerning the investigation on the response of the piezoionic pullulan sensor to deformation, the suitable setup, described in the characterization section and showed in Scheme 1, along with a corresponding picture, was devised for a comprehensive analysis of the mechanoelectrical transduction behavior of the developed devices.

**Figure 6:**
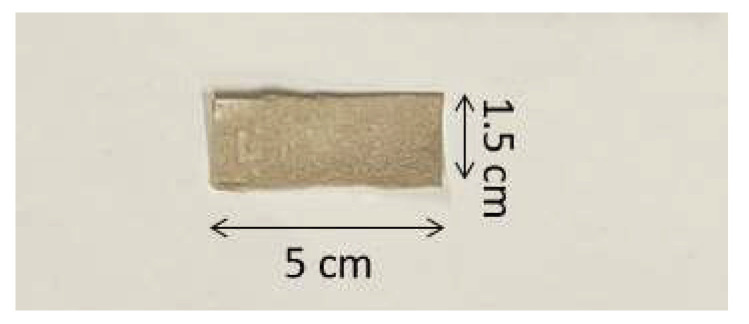
Picture of the pullulan-based sensor.

The setup included a 5 cm long and 1.5 cm wide pullulan-based sensor (Figure 6).

**Figure 7:**
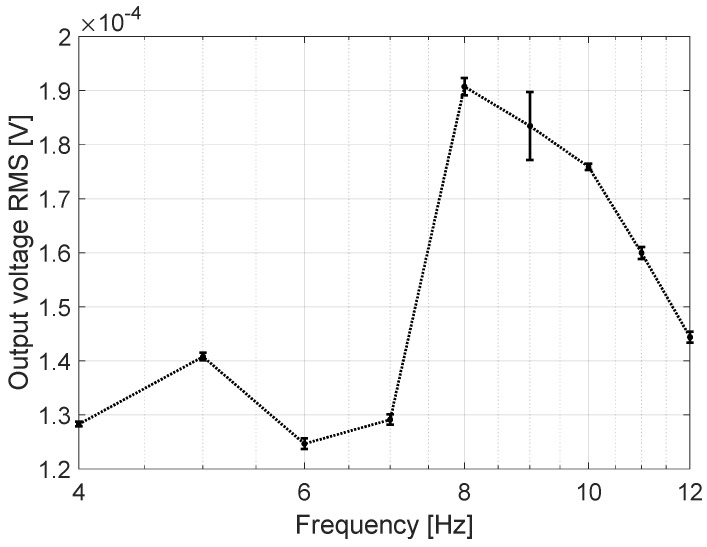
Output voltage of the pullulan-based sensor as a function of the frequency in the presence of a sinusoidal mechanical excitation. The dots have been connected for clarity.

Figure 7 shows the root mean squared (RMS) output voltage of the sensor relative to the excitation frequency. Using the Keysight Technologies 33220A function waveform generator, a sinusoidal waveform of fixed amplitude and varying frequency was applied. The vibration level, approximately 6 m/s^2^, was measured via the feedback accelerometer PCB333B40-SN51174.

Specifically, a sinusoidal excitation ranging from 4 to 12 Hz was driven via the shaker. The Agilent Infiniium MSO9064A oscilloscope was used to measure the mean output voltage and standard deviation at each frequency. Notably, a prominent peak at 8 Hz aligned with the device’s mechanical resonant frequency.

**Figure 8:**
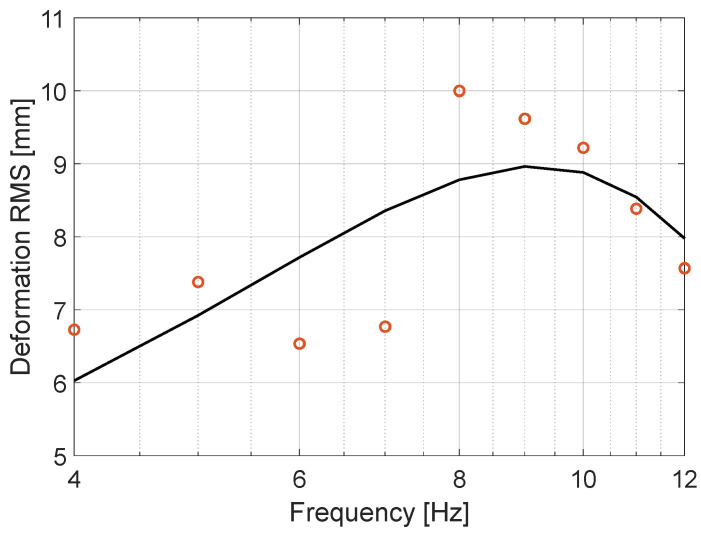
Deformation of the sensor as a function of the frequency in the presence of a sinusoidal mechanical excitation. A gaussian fitting was addressed through a curve-fitting tool.

Figure 8 shows the RMS deformation of the device concerning the sinusoidal excitation signal. The deformation measurements relied on the two Baumer 12U6460/S35A laser sensors, calculating the difference between their respective displacements. An observed maximum deformation amplitude of approximately 1 cm coincided with the mechanical resonant frequency of the device.

Additionally, a curve-fitting tool was utilized, resulting in the following Gaussian fitting:

(1) $$f\left(x\right)=a_{1}e^{-{\left(\frac{x-b_{1}}{c_{1}}\right)}^{2}}$$

where *x* represents the frequency of the vibrational sinusoidal signal, *a*_1_ = 8.97, *b*_1_ = 9.19, and *c*_1_ = 8.23. The graph includes the measurements together with the curve fitting.

**Figure 9:**
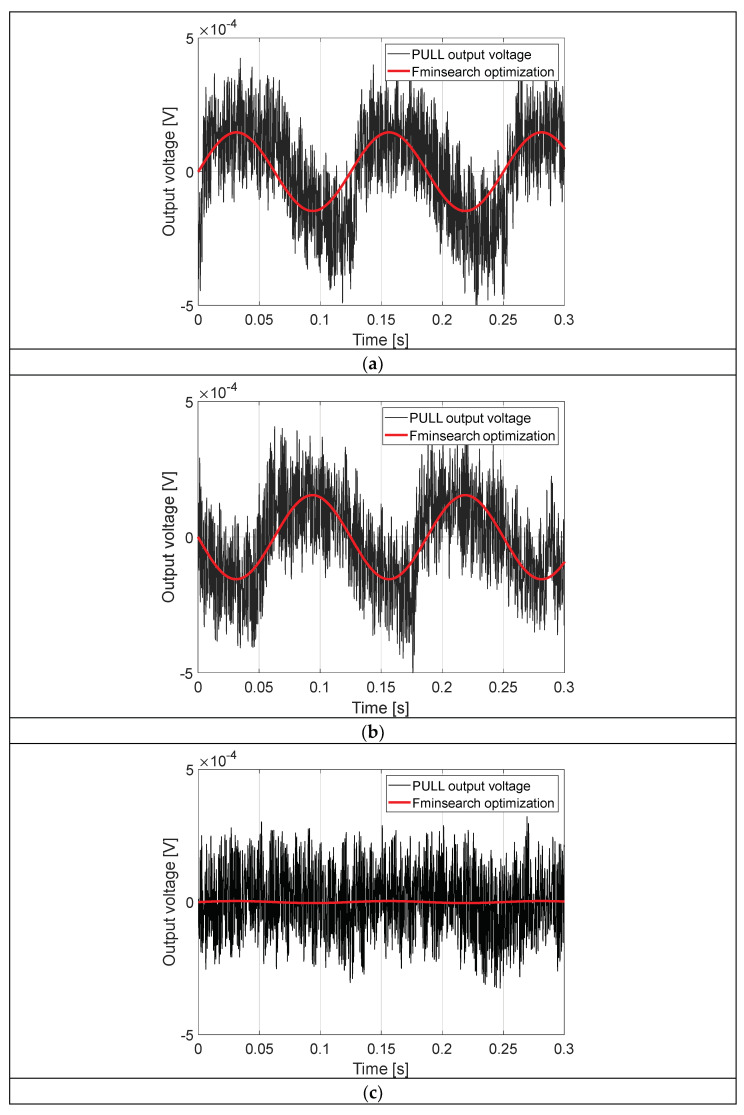
Output voltage of the sensor as a function of the time at (**a**) 10 mm, (**b**) 8 mm, and (**c**) 3.5 mm of deformation, respectively. The graphs include the approximation with a sinusoidal waveform, obtained by using the MATLAB^®^ function *fmninsearch*.

A time domain study was also conducted, examining the sensor’s output voltage over time (refer to Figure 9). Specifically, the focus was on the sensor’s output voltage under resonant conditions, with variations in the applied deformation amplitude induced by the shaker. This amplitude was estimated based on the difference in displacements measured by the output of the laser sensors at the tip and anchor.

The graph depicts the output voltage juxtaposed with a sinusoidal waveform derived through a MATLAB^®^ optimization process. This process employed a nonlinear programming solver aimed at minimizing a quadratic deviation problem, utilizing the fminsearch function. The waveforms on the graph exhibit a noticeable attenuation of the output signal.

**Figure 10:**
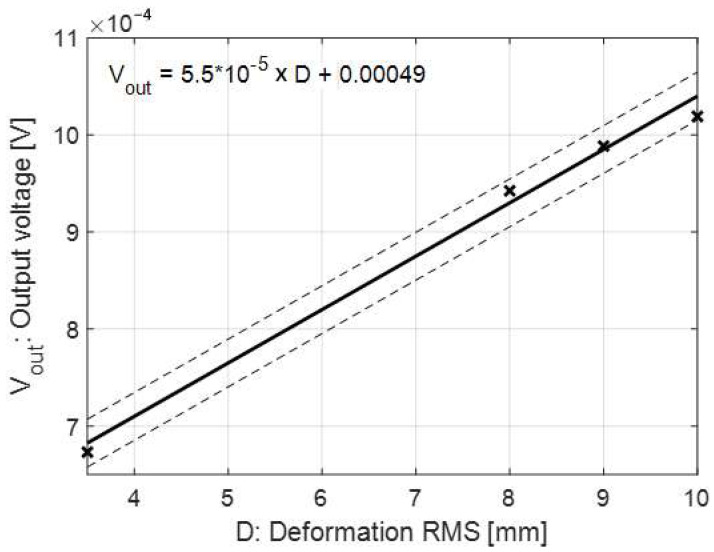
The output voltage of the sensor evaluated in the presence of a sinusoidal vibration at 8 Hz as a function of the applied RMS deformation. Cross marks represent experimental results, dashed lines represent standard uncertainties derived from 10 repetitions.

Sensor characterization was executed at the resonant frequency, manipulating the amplitude of the input sinusoidal signal while estimating the pullulan-based sensor deformation via the output of the laser sensors. Figure 10 portrays the device’s output voltage for various RMS deformation values, featuring both mean values and corresponding experimental standard uncertainties derived from 10 repetitions. A sensitivity of about 5.5 × 10^−5^ V/mm has been estimated with a resolution of about 1 mm. The following transduction function describes the characteristic curve of the device:

(2) $$V_{out}=S\cdot D+K=5.5\cdot 10^{-5}D+0.00049$$

Thus, *V_out_* is the generated output voltage in presence of an applied deformation (D). The term S is the sensitivity of the device while an additive constant, K, was also considered.

## 4. Conclusions

In conclusion, by combining a water-soluble biopolymer and a water-soluble IL, it is possible to produce a vibration sensor that easily degrades, thereby falling within the field of transient electronics. The three-layer sensor, having a core made of a composite PUL/EMIMBF_4_ coated on both sides with a conductive silver-based varnish, operates by leveraging the piezoionic effect induced by the presence of the IL. The PUL–IL interaction determines a slight decrease of the thermal stability of both of the components and a lower conservative modulus in the 1 to 50 Hz frequency range (approximately 2 × 10^7^ Pa compared to the value of about 1 × 10^8^ Pa of the pure PUL membrane). Operating as a vibration sensor, it identifies resonance at roughly 8 Hz. The conducted time domain study focused on the sensor’s output voltage under resonant conditions which exhibit a noticeable attenuation of the output signal going from 10 mm, 8 mm, and 3.5 mm of deformation, respectively. Operating as a vibration sensor, it offers a sensitivity of about 5.5 × 10^−5^ V/mm and a resolution of about 1 mm.

The future development of this study involves the evaluation of the impact of ionic liquid concentration in the membrane on the viscoelastic behavior and sensor performance and the realization of devices based on biopolymers with piezoionic components derived from biological sources in combination with biodegradable electrodes.

## Figures and Tables

**Table 1:** Measurements of the storage modulus (E′) and the loss modulus (E″) of PUL membrane, PUL/EMIMBF_4_, and PUL/EMIMBF_4_/Ag.

		PUL	PUL/EMIMBF_4_	PUL/EMIMBF_4_/Ag
E′ (Pa)	1 Hz	1.0 × 10^8^	2.5 × 10^7^	1.9 × 10^7^
	10 Hz	1.1 × 10^8^	2.8 × 10^7^	2.2 × 10^7^
	50 Hz	1.1 × 10^8^	3.1 × 10^7^	2.9 × 10^7^
E″ (Pa)	1 Hz	4.0 × 10^6^	1.9 × 10^6^	1.6 × 10^6^
	10 Hz	4.5 × 10^6^	2.6 × 10^6^	2.3 × 10^6^
	50 Hz	4.6 × 10^6^	6.9 × 10^6^	7.1 × 10^6^

## Data Availability

The data presented in this study are available on request from the corresponding author. The data are not publicly available due to privacy.
